# Patients With Ankylosing Spondylitis Are Associated With High Risk of Fibromyalgia: A Nationwide Population-Based Cohort Study

**DOI:** 10.3389/fmed.2021.618594

**Published:** 2021-03-11

**Authors:** Shuo-Yan Gau, Yung-Heng Lee, Hsi-Kai Tsou, Jing-Yang Huang, Xinpeng Chen, Zhizhong Ye, James Cheng-Chung Wei

**Affiliations:** ^1^School of Medicine, Chung Shan Medical University, Taichung, Taiwan; ^2^Department of Senior Services Industry Management, Minghsin University of Science and Technology, Hsinchu, Taiwan; ^3^Department of Recreation and Sport Management, Shu-Te University, Kaohsiung, Taiwan; ^4^Department of Orthopedics, Cishan Hospital, Ministry of Health and Welfare, Kaohsiung, Taiwan; ^5^Functional Neurosurgery Division, Neurological Institute, Taichung Veterans General Hospital, Taichung, Taiwan; ^6^Department of Rehabilitation, Jen-Teh Junior College of Medicine, Nursing and Management, Miaoli County, Taiwan; ^7^Center for Health Data Science Chung Shan Medical University Hospital, Taichung, Taiwan; ^8^Institute of Medicine, Chung Shan Medical University, Taichung, Taiwan; ^9^Department of Rheumatology, Shenzhen Futian Hospital for Rheumatic Diseases, Shenzhen, China; ^10^Department of Allergy, Immunology and Rheumatology, Chung Shan Medical University Hospital, Taichung, Taiwan; ^11^Graduate Institute of Integrated Medicine, China Medical University, Taichung, Taiwan

**Keywords:** ankylosing spondylitis, fibromyalgia, cohort, population-based study, NHIRD

## Abstract

**Objectives:** The main purpose of this retrospective cohort study was to provide an evaluation of Ankylosing spondylitis (AS) patients' fibromyalgia risk in different age and sex subgroups by analyzing large study samples.

**Methods:** Datasets from the National Taiwan Insurance Research Database (NHIRD) were retrieved in this retrospective cohort study. This study was approved by the Institutional Review Board of Chung Shan Medical University (IRB permit number CS15134). Within the Longitudinal Health Insurance Database (LHID), and the subset of NHIRD, we identified AS patients to explore the risk of further fibromyalgia. The exposure cohort included patients with newly-diagnosed AS (ICD-9-CM:720.0) during 2000–2013. After 1:4 age-sex matching and 1:2 propensity score matching, and adjusting potential confounders, individuals without AS were identified as a comparison cohort. The adjusted hazard ratio of subsequent development of fibromyalgia in people with AS was evaluated. Further stratification analyses of different ages and genders were then undertaken to validate the results.

**Results:** In total, 17 088 individuals were included in the present study, including 5,696 patients with AS and 11,392 individuals without AS. Respective incidence rates (per 1,000 person-months) of fibromyalgia was 0.52 (95% CI, 0.46–0.59) in the AS cohort and 0.39 (95% CI, 0.35–0.44) in the non-AS cohort. Compared with the non-AS cohort, aHR of developing fibromyalgia was 1.32 (95% CI, 1.12–1.55) in people with AS. This association was consistent in both statistical models of 1:4 age–sex matching and 1:2 propensity score matching.

**Conclusion:** Patients with AS were associated with a higher risk of fibromyalgia, especially those over 65 years old. In managing patients with AS, clinicians should be aware of this association, which could impact diagnosis, disease activity evaluation, and treatment.

## Introduction

Ankylosing spondylitis (AS) causes chronic inflammatory back pain in 70–80% of patients (1), which massively decreases the life quality of patients (2). According to epidemiological data, in Taiwan, the incidence of AS is 24.2 (per 100,000 person-years), and the prevalence is 96.9 (per 100,000 people). People between ages 20–29 years old showed the highest prevalence and incidence rate (3). Spinal inflammation and structural damage might lead to loss of spinal mobility in patients with AS (4).

As a neuropathic pain syndrome, fibromyalgia contributes to multifocal pain. Because of the dysregulated function in the central nervous system and amplification of the sense of pain, symptoms such as musculoskeletal pain, visceral pain, and chronic headaches are often observed in patients (5, 6). In fibromyalgia patients, changes in the number of inflammatory cytokines have been widely reported, and the role of neurogenic inflammation has been thought to play a potential role between inflammatory disorder and the development of fibromyalgia (7). Clinically, fibromyalgia is common in patients with histories of inflammatory diseases with symptoms of chronic pain, including Sjögren syndrome (8), systemic lupus erythematosus (SLE) (9, 10), or rheumatoid arthritis (RA) (6). Accordingly, the term *Secondary Fibromyalgia* is used to describe fibromyalgia based on existing rheumatological or other inflammatory disorders (7).

Fibromyalgia is thought to be a comorbidity of inflammatory rheumatic diseases with reported high prevalence; however, for ankylosing spondylitis, the evidence is lacking and controversial. Previous studies have reported the high prevalence of fibromyalgia between people with AS (11, 12). In contrast, another previous study also stated that compared with normal people, the incidence of fibromyalgia did not increase in patients with AS (13). Due to the lack of large-scale studies evaluating the relation between AS and fibromyalgia, this study aimed to determine whether AS increases the risk of fibromyalgia in a long-term population-based cohort database.

## Materials and Methods

### Data Source

Datasets from the Longitudinal Health Insurance Database (LHID) 2000 were retrieved in this retrospective cohort study. LHID 2000 contains one million individuals who were randomly sampled from the 2000 Registry for Beneficiaries and comprised their claim data from the National Health Insurance Research Database (NHIRD). In the dataset, diagnosis records, medical claims and prescriptions during hospitalizations and ambulatory care were used for study analysis. The International classification of diseases, ninth revision, clinical modification (ICD-9-CM code) was utilized in NHIRD to provide diagnosis records. Information about patients or caregivers was anonymized before data accumulation to protect privacy. This study was approved by the Institutional Review Board of Chung Shan Medical University Hospital.

### Identification of Patients With AS

Patients with AS diagnosis (ICD-9-CM: 720.0) from 1997 to 2013 were identified. To ensure accuracy, AS patients were defined as at least one inpatient diagnosis or two outpatient visits, excluding people with only one inpatient visit for disease diagnosis or treatment.

**Figure 1:**
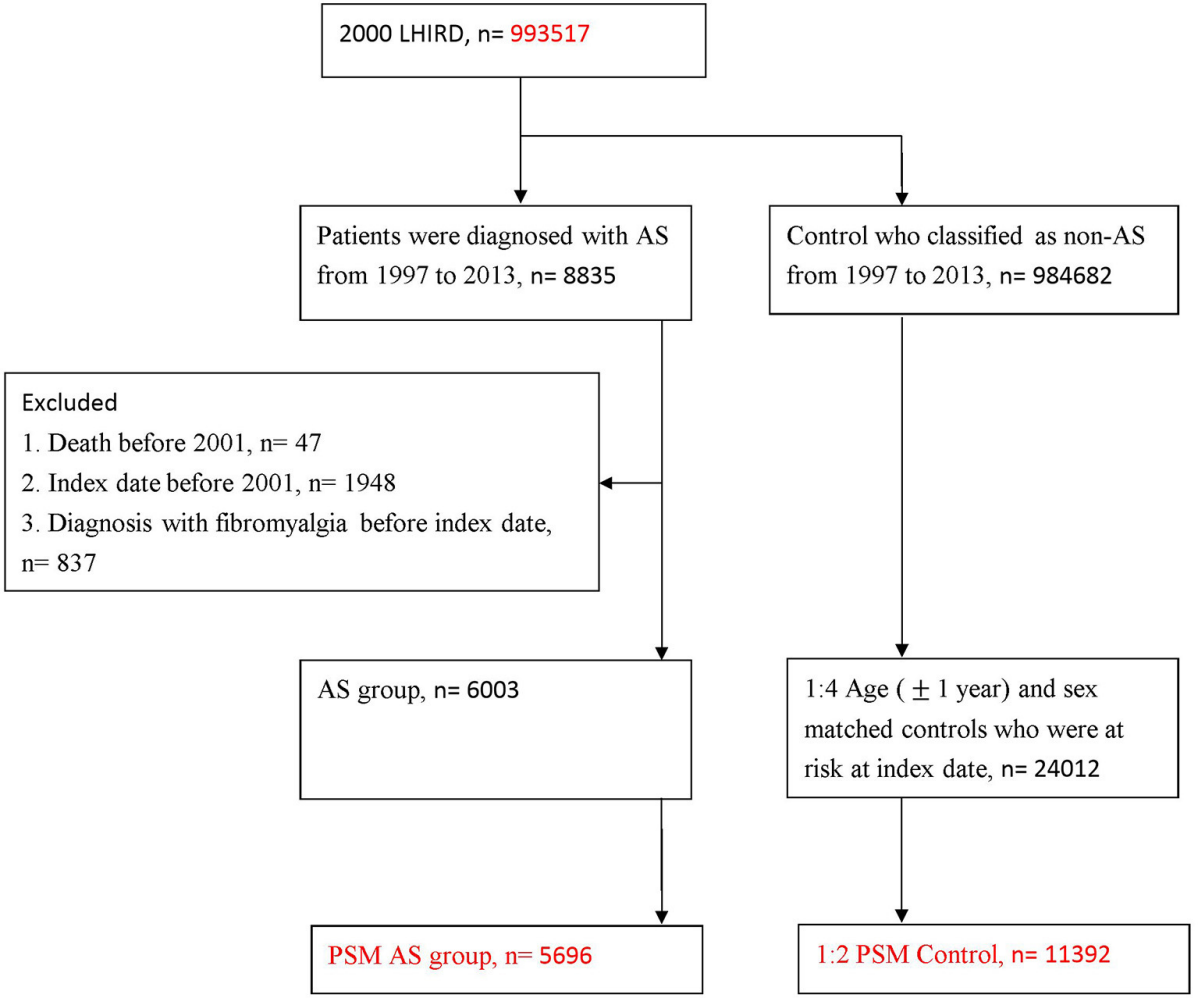
Study flow chart of patient selection.

Furthermore, for improving the validity of AS diagnosis, we defined AS in those who received non-steroidal anti-inflammatory drugs (NSAIDs) or Sulfasalazine within 1 month following the AS diagnosis. The first date of AS diagnosis was defined as the index date in the AS cohort. In the present dataset, 8,835 patients with AS corresponding to the definition were included in the study. Individuals with (1) death before 2001 (*n* = 47), (2) index date before 2001 (*n* = 1,948) and (3) diagnosis of fibromyalgia before index date (*n* = 837) were excluded. This left 6,003 patients with AS for analysis. To deal with the potential confounding effect, and balance the baseline characteristics, we conducted propensity score matched (PSM) analysis. Finally, after PSM, 5,696 patients with AS were included (Figure 1).

### Selection of Comparison Cohort

For the non-AS cohort, we included patients who had never been diagnosed with AS from 1997 to 2013. The AS cohort was matched with the non-AS cohort to give a start point at a ratio of 1:4 ratio by age and sex. To prevent potential confounding bias, the AS cohort was matched with the non-AS cohort by PSM with a greedy matching algorithm (14) at a 1:2 ratio. The propensity score of AS probability was evaluated by logistic regression model with predictors including sex, age, income, urbanization, length of hospital stay within 1 year before the index date, comorbidities (including rheumatoid arthritis, hypertension, diabetes mellitus, hyperlipidemia, chronic kidney disease, Chronic Obstruction Pulmonary Disease (COPD), chronic liver diseases, gout, and depression). Standardized differences were used to evaluate the balance covariates in baseline between cohorts.

### Identification of Subsequent Fibromyalgia

The definition of subsequent fibromyalgia was based on fibromyalgia diagnosis (ICD-9: 729.1) after the index date, with at least one inpatient diagnosis or two outpatient visits. According to previous articles, this diagnosis was of accuracy and validity (15, 16). As additional criteria, individuals were required to receive drugs for fibromyalgia (including selective serotonin reuptake inhibitors (SSRI)/serotonin and norepinephrine reuptake inhibitors (SNRI), Pregabalin, Gabapentin, Amitriptyline, Tramadol, Cyclobenzaprine, or Tizanidin) within 30 days after the fibromyalgia diagnosis. All individuals were followed up at the start of the index date until the first occurrence of fibromyalgia, death, or December 2013.

### Statistical Analysis

Univariate Poisson modeling was used to calculate crude relative risk, 95% confidence interval (95% CI), and incidence rate. The difference in baseline characteristics between AS and the non-AS cohort was evaluated by absolute standardized difference (ASD) (17). With ASD <0.10, covariate was regarded as a small difference. The association between study factors in the study and fibromyalgia events was determined by the adjusted hazard ratio (aHR) with 95% CI Kaplan–Meier methods were used to estimate the cumulative probability of fibromyalgia. A Log-rank test was performed to compare the difference in cumulative probabilities between the two cohorts. We performed age- and sex- subgroup analyses to explore the potential interactions between AS and subgroup variables. All data were analyzed by SAS (version 9.4; SAS Institute, Cary, NC, USA) software.

### Role of the Funding Source

The Dry Lab Team of Chung Shan Medical University helped this study with access to the database and technique support in data analysis and interpretation.

## Results

In total, 8,835 patients with AS diagnosis and who had been treated with NSAIDs or Sulfasalazine were included. After excluding cases that were not at risk at baseline there were 6,003 patients for analysis. Finally, through 1:2 PSM, a total of 5,696 patients and 11,392 matched non-AS cohort were included (Figure 1).

In the distribution of baseline characteristics, the sex ratio (F:M) of people with AS was 42:58 before PSM. AS patients stayed for a longer number of days in hospital compared with the non-AS cohort, with the ASD > 0.10 before PSM. The proportions of comorbidities (such as RA, hypertension, hyperlipidemia, chronic liver diseases, and gout) and medications (including systemic corticosteroids, NSAIDs, hydroxychloroquine, methotrexate, sulfasalazine) were non-balance and higher in AS patients before matching. Through PSM, the baseline characteristics became more homogeneous between AS and non-AS groups, and ASDs were < 0.1 in all variables (excluding medication variables) (Table 1).

**Table 1:** Baseline characteristics among study groups.

	**Before PSM (1:4 age–sex matching)**			**After 1:2 PSM**		
	**Non-AS *n* = 24,012**	**AS*n*= 6,003**	**ASD**	**Non-AS*n* = 11,392**	**AS *n* = 5,696**	**ASD**
Sex			0.000			0.027
Female	10,024 (41.75%)	2,506 (41.75%)		4,689 (41.16%)	2,330 (40.91%)	
Male	13,988 (58.25%)	3,497 (58.25%)		6,703 (58.84%)	3,366 (59.09%)	
Age			0.004			0.008
<30	6,305 (26.26%)	1,566 (26.09%)		2,980 (26.16%)	1,511 (26.53%)	
30–45	6,931 (28.86%)	1,754 (29.22%)		3,267 (28.68%)	1,666 (29.25%)	
45–65	6,983 (29.08%)	1,734 (28.89%)		3,274 (28.74%)	1,609 (28.25%)	
≥65	3,793 (15.80%)	949 (15.81%)		1,871 (16.42%)	910 (15.98%)	
Urbanization			0.076			0.022
Urban	14,542 (60.56%)	3,509 (58.45%)		6,534 (57.36%)	3,319 (58.27%)	
Sub-urban	7,182 (29.91%)	1,870 (31.15%)		3,650 (32.04%)	1,783 (31.30%)	
Rural	2,288 (9.53%)	624 (10.39%)		1,208 (10.60%)	594 (10.43%)	
Low income	145 (0.60%)	24 (0.40%)	0.029	47 (0.41%)	24 (0.42%)	0.001
Length of hospital stays^†^			0.136			0.000
0 day	22,146 (92.23%)	5,292 (88.16%)		10,114 (88.78%)	5,042 (88.52%)	
1–6 days	1,082 (4.51%)	4,35 (7.25%)		757 (6.65%)	402 (7.06%)	
≥7 days	784 (3.27%)	276 (4.60%)		521 (4.57%)	252 (4.42%)	
Co-morbidity^†^						
RA	127 (0.53%)	299 (4.98%)	0.275	5 (0.04%)	3 (0.05%)	0.004
Hypertension	3,450 (14.37%)	1,160 (19.32%)	0.133	2,090 (18.35%)	1,097 (19.26%)	0.023
Diabetes mellitus	1,604 (6.68%)	518 (8.63%)	0.073	9,66 (8.48%)	493 (8.66%)	0.006
Hyperlipidemia	1,777 (7.40%)	638 (10.63%)	0.113	1,101 (9.66%)	596 (10.46%)	0.027
CKD	188 (0.78%)	60 (1.00%)	0.023	108 (0.95%)	55 (0.97%)	0.002
COPD	1,202 (5.01%)	416 (6.93%)	0.081	759 (6.66%)	391 (6.86%)	0.008
Chronic liver diseases	1,049 (4.37%)	439 (7.31%)	0.126	703 (6.17%)	412 (7.23%)	0.042
Gout	935 (3.89%)	506 (8.43%)	0.189	708 (6.21%)	440 (7.72%)	0.059
Depression	1,571 (6.54%)	727 (12.11%)	0.192	1,124 (9.87%)	672 (11.80%)	0.062
Medication^†^						
Systemic corticosteroids	4,913 (20.46%)	2,339 (38.96%)	0.413	3,850 (33.80%)	2,145 (37.66%)	0.081
NSIADs	14,298 (59.55%)	5,874 (97.85%)	1.059	11,135 (97.74%)	5,567 (97.74%)	0.001
Hydroxychloroquine	50 (0.21%)	169 (2.82%)	0.215	21 (0.18%)	102 (1.79%)	0.163
Methotrexate	34 (0.14%)	107 (1.78%)	0.169	13 (0.11%)	63 (1.11%)	0.128
Sulfasalazine	30 (0.12%)	1,172 (19.52%)	0.689	7 (0.06%)	1,027 (18.03%)	0.660

† *The length of hospital stays, co-morbidity, use of medications were identified within 1 year before index date*.

**Figure 2:**
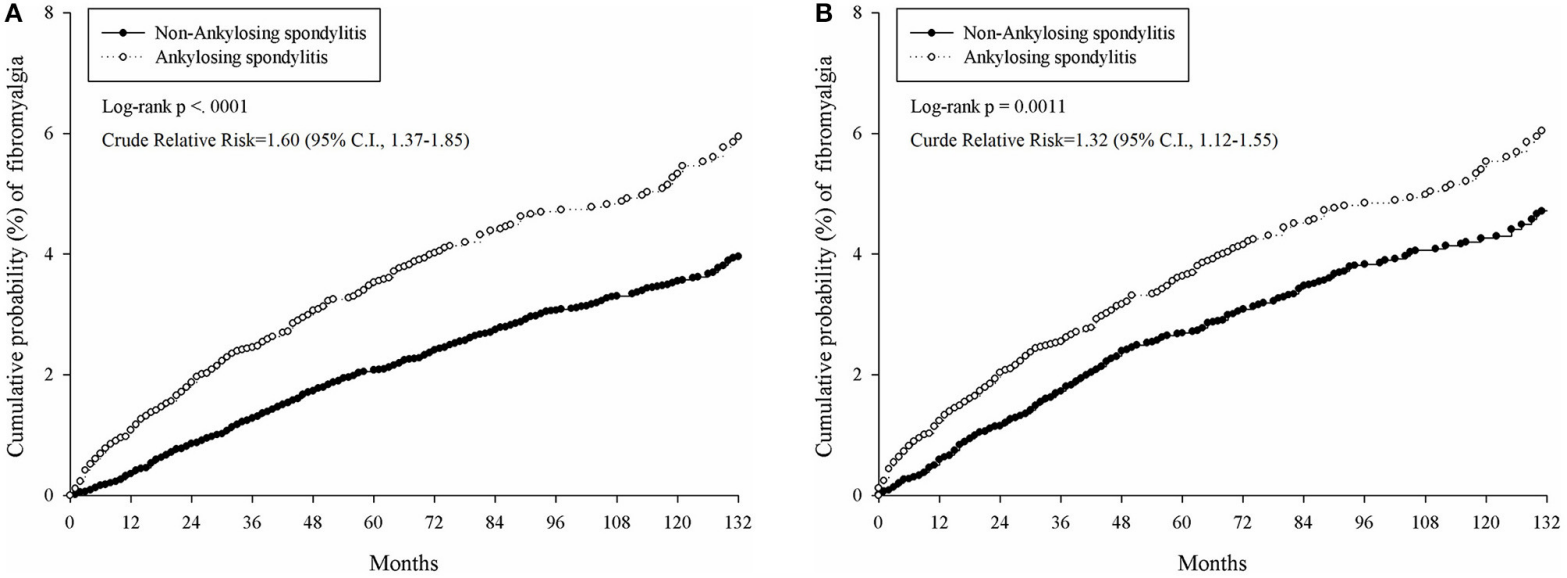
Comparison of cumulative probability of fibromyalgia in patients with and without AS under different models. **(A)** Cumulative probability of fibromyalgia in AS patients and 1:4 age–sex matched controls who were at risk at index date. **(B)** Cumulative probability of fibromyalgia in AS patients and 1:2 PSM control.

The cumulative probability of developing fibromyalgia was presented in Table 2 and Figure 2. The incidence rate of fibromyalgia in AS patients was 0.52 per 1,000 person months (95% CI, 0.46–0.59); whereas in the non-AS cohort, the incidence was 0.39 per 1,000 person months (95% CI, 0.35–0.44). The AS group showed a higher risk of developing fibromyalgia than the non-AS group (crude relative risk = 1.32, log-rank test, *p* = 0.0011, 95% CI, 1.12–1.55).

**Table 2:** Incidence of fibromyalgia in age–sex matched group.

	**Before PSM (1:4 age–sex matching)**		**After 1:2 PSM**	
	**Non-AS *n* = 24,012**	**AS*n* = 6,003**	**Non-AS *n*= 11,392**	**AS*n* = 5,696**
Follow up person months	1,913,078	471,482	905,332	447,988
New fibromyalgia case	607	239	356	232
Incidence rate^*^(95% CI)	0.32 (0.29–0.34)	0.51 (0.45–0.58)	0.39 (0.35–0.44)	0.52 (0.46–0.59)
Crude Relative risk (95% CI)	Reference	1.60 (1.37–1.85)	Reference	1.32 (1.12–1.55)

* *Incidence rate, per 1,000 person-months*.

Table 3 indicates the aHR of developing fibromyalgia in specific characteristics. In the univariate model in age and sex matched population, patients with AS had a significantly increased aHR (1.60, 95% CI, 1.37–1.85) of fibromyalgia. Furthermore, the risk factors also involved age, urbanization, co-morbidity with diabetes mellitus and gout. The use of NSAIDs also influences the occurrence of fibromyalgia. In the second model, population after PSM, the aHR of AS patients developing fibromyalgia was 1.39 (95% CI, 1.16–1.67).

**Table 3:** Cox proportional hazard regression for estimation of adjusted hazard ratios on fibromyalgia.

	**aHR (95% CI)**		
	**Age–sex matched population**		**PSM population**
**Variable**	**Univariate**	**Multiple**	**Conditional cox**
AS	1.60 (1.37–1.85)	1.28 (1.09–1.50)	1.39 (1.16–1.67)
Sex of male	0.75 (0.66–0.86)	0.93 (0.81–1.07)	
Age			
<30	0.62 (0.50–0.78)	0.65 (0.52–0.81)	
30–45	Reference	Reference	
45–65	1.46 (1.22–1.74)	1.34 (1.12–1.61)	
≥65	2.24 (1.85–2.70)	1.89 (1.53–2.34)	
Urbanization			
Urban	Reference	Reference	
Sub-urban	1.17 (1.00–1.36)	1.11 (0.95–1.29)	
Rural	1.89 (1.55–2.29)	1.64 (1.35–2.00)	
Length of hospital stays^†^, day			
0	Reference	Reference	
1–6	1.24 (0.94–1.65)	0.94 (0.70–1.25)	
≥7	1.33 (0.94–1.89)	0.82 (0.57–1.17)	
Co-morbidity^†^			
RA	1.01 (0.57–1.79)	0.67 (0.38–1.19)	
Hypertension	1.82 (1.55–2.14)	0.91 (0.75–1.11)	
Diabetes mellitus	2.04 (1.66–2.52)	1.28 (1.01–1.62)	
Hyperlipidemia	1.89 (1.54–2.32)	1.07 (0.85–1.35)	
CKD	1.81 (0.94–3.48)	1.03 (0.53–2.01)	
COPD	1.40 (1.07–1.82)	0.91 (0.69–1.19)	
Chronic liver diseases	1.61 (1.25–2.07)	1.13 (0.88–1.47)	
Gout	2.03 (1.60–2.57)	1.45 (1.13–1.86)	
Depression	2.48 (2.07–2.98)	1.80 (1.49–2.18)	
Use of NSIADs^†^	2.07 (1.75–2.46)	1.70 (1.42–2.04)	

† *The length of hospital stays, co-morbidity, use of medication were identified within 1 year before index date*.

After sensitivity analysis, we found that female patients with AS showed a higher risk of developing fibromyalgia (aHR = 1.32, 95% CI, 1.05–1.66) compared with the non-AS cohort. Similarly, a higher tendency of fibromyalgia was also observed in male AS patients (aHR = 1.24, 95% CI, 1.00–1.55). Furthermore, for AS patients older than 65 years, a higher risk of developing fibromyalgia was statistically significant (aHR = 1.55, 95% CI, 1.15–2.09). However, we did not find any interaction effect between AS with sex or age in risk of fibromyalgia (Table 4).

**Table 4:** Sub-group analysis in age–sex matched population.

	**Non-AS**		**AS**		
**Sub-group**	**Sample size**	Incidence rate^*^ (95% CI)	**Sample size**	Incidence rate^*^ (95% CI)	**aHR+ (95% CI)**
Sex					
Female	10,024	0.37 (0.33–0.42)	2,506	0.58 (0.48–0.70)	1.32 (1.05–1.66)
Male	13,988	0.28 (0.25–0.31)	3,497	0.46 (0.38–0.54)	1.24 (1.00–1.55)
Age					
<30	6,305	0.16 (0.13–0.20)	1,566	0.26 (0.19–0.36)	1.12 (0.74–1.70)
30–45	6,931	0.26 (0.22–0.31)	1,754	0.43 (0.33–0.55)	1.28 (0.93–1.76)
45–65	6,983	0.40 (0.35–0.46)	1,734	0.57 (0.46–0.72)	1.17 (0.88–1.54)
≥65	3,793	0.58 (0.50–0.68)	949	1.07 (0.85–1.37)	1.55 (1.15–2.09)
Urbanization					
Urban	14,542	0.28 (0.26–0.32)	3,509	0.43 (0.36–0.52)	1.22 (0.98–1.52)
Sub-urban	7,182	0.32 (0.27–0.37)	1,870	0.55 (0.44–0.68)	1.36 (1.03–1.79)
Rural	2,288	0.53 (0.44–0.65)	624	0.82 (0.60–1.12)	1.47 (0.98–2.20)
Length of hospital stays					
0	22,146	0.31 (0.28–0.34)	5,292	0.51 (0.44–0.58)	1.30 (1.10–1.54)
1–6	1,082	0.39 (0.28–0.55)	435	0.52 (0.33–0.83)	1.29 (0.71–2.34)
≥7	784	0.47 (0.31–0.70)	276	0.48 (0.26–0.89)	1.01 (0.47–2.20)

* Means per 1000 person-months;

*^+^(beside the column of aHR) means adjusted for demographic variables, length of hospital stay, co-morbidities, and use of NSAIDs at baseline*.

## Discussion

This study reports the possible association between AS patients and the occurrence of future secondary fibromyalgia by utilizing a robust nationwide longitudinal population-based database. Patients with AS are associated with a higher risk of subsequent fibromyalgia, compared with a matched non-AS cohort. Furthermore, both male and female subgroups have a higher risk of developing fibromyalgia.

Pain is a crucial criterion for AS classification (2). Though intermittent at onset, AS-associated pain will gradually become persistent (18). Both inflammatory pain and neuropathic pain are identified in AS (19, 20). In inflammation states, especially in chronic pain, immune cells assemble around nociceptors in nerve terminals (21). Nociceptors are thus sensitized, having a higher tendency stimulated by inflammatory mediators (18, 22). Observed in arthritis patients including AS, central sensitization causes type 1 T helper cells (TH1 cells) (23), which are induced to migrate into the spinal cord and lead to pain hypersensitivity (24, 25). Neuroinflammation has a strong relation with fibromyalgia. Chronic systemic inflammation might lead to neuroinflammation and is considered to play a role in the pathogenesis in fibromyalgia, with antibodies such Interleukin-1Ra or Interleukin-6 elevated in patients' serum (7, 26–28). Rheumatic disorders including RA, SLE, and inflammatory bowel disease were regarded as associating with a higher prevalence of fibromyalgia because of painful conditions spread throughout the musculoskeletal system (7, 16, 29, 30). The previous article also reported the higher rate of fibromyalgia between people with AS, especially in women (11, 12, 31). Additionally, environmental factors, including chronic acute pain are risk factors for triggering fibromyalgia (5). Central sensitization is thought to result in fibromyalgia and involves a decrease in pain inhibiting function in the central nerve system (7, 24, 32). Both AS and fibromyalgia are associated with central sensitization. Furthermore, AS has a strong relation with systemic inflammation and chronic pain, which are also risk factors of fibromyalgia; however, the actual association of AS and subsequent fibromyalgia still lacks certain population-based evidence. Correspondingly, our study demonstrated that people with AS are associated with a greater risk of future fibromyalgia.

Men are more likely affected by AS than women (4). Accordingly, in our study group, the male subgroup was larger than the female subgroup in AS patients. Previous studies indicate that compared with men, fibromyalgia showed higher prevalence in women, and women with AS are considered to have a higher risk of developing fibromyalgia (11, 31, 33). In the present study, both male (aHR = 1.24, 95% CI, 1.00–1.55) and female (aHR=1.32, 95% CI, 1.05–1.66) participants showed a higher tendency of developing fibromyalgia, and the tendency for both was statistically significant. The risk of female patients developing fibromyalgia is slightly higher than in male patients. Although the difference was not statically significant, the result supported the conclusions of previous research.

This study offers insights into the epidemiological relationship between AS and the potential risk of fibromyalgia. Through using this database, the association between diseases could be observed on a longer time scale. However, some limitations should be addressed. First, in the system of NHIRD, we could only identify diseases through ICD-9-CM codes, related examinations, drugs, or special therapies, as further information for identification was not available. However, compared with other prospective medical records, ICD-9-CM codes, as administrative data, might not precisely define diseases on a prospective medical record and could result in potential misclassification of diseases.

As for the definition of AS, though the related ICD-9-CM codes under NHIRD have not been validated by further studies, the definition we chose for AS in our study (ICD9-CM 720.0, with 1 inpatient or 2 outpatient visits, above) through ICD-9-CM codes has been applied in a previous NHIRD study (34). As for ensuring the accuracy of definition for fibromyalgia, the definition we adopted in the present study (ICD-9-CM code 729.1) was applied in previous literature (15, 16). Moreover, we included eight drugs for fibromyalgia treatment as an extra defining factor of fibromyalgia to minimize these disadvantages. Despite the critical role depression plays within patients with fibromyalgia, as potentially patients have both diseases, the risk of confusing drug utilization between these two diseases is unlikely. Nonetheless, since clinically these drugs are widely prescribed to fibromyalgia patients, compared with merely using ICD-9 CM code to define fibromyalgia, we believe that for the patients with no depression, and there were plenty of in our study group, adding additional criteria for drug prescriptions could make the definition more precise. Second, we were unable to include information on the potential influence of risk factors that make patients susceptible to fibromyalgia, including infection status, psychological, and behavioral issues as data on these factors were unavailable in NHIRD. However, the present study did consider the influence of medications for AS and comorbidities of AS. Third, potential differential misclassification does exist. For instance, during clinic visits, AS patients might be more likely asked about fibromyalgia rather than the healthy non-AS cohort, which might lead to possible information bias. Fourth, since patients with rheumatic diseases might have a higher tendency to be asked about fibromyalgia, surveillance bias does exist. Future studies should validate whether risk factors of fibromyalgia in AS patients will contribute to differences in subsequent fibromyalgia development.

In conclusion, this population-based cohort study indicated that people with AS have a higher risk of developing fibromyalgia. When managing patients with AS or fibromyalgia, clinicians should be alert of this possible association.

## Data Availability Statement

Datasets from the Longitudinal Health Insurance Database (LHID) 2000 were retrieved in this retrospective cohort study, and the data are available from the Taiwan National Health Insurance (NHI) Bureau. The data are not publicly available because of legal restrictions regarding the “Personal Information Protection Act” in Taiwan. Requests to access the datasets should be directed to https://dep.mohw.gov.tw/DOS/cp-2516-3591-113.html.

## Ethics Statement

The studies involving human participants were reviewed and approved by the Institutional Review Board of Chung Shan Medical University (IRB permit number CS15134). Written informed consent to participate in this study was provided by the participants' legal guardian/next of kin.

## Author Contributions

JW was responsibility for the integrity and accuracy of data and analysis. S-YG, JW, J-YH, Y-HL, H-KT, XC, and ZY were involved in study conception and design. JW and J-YH undertook the acquisition of data. S-YG, JW, Y-HL, H-KT, XC, and ZY performed analysis and interpretation of data. S-YG, JW, and J-YH wrote and prepared the original draft of the manuscript. JW, J-YH, and S-YG verified underlying data. All authors were involved in drafting and revising the article and approved the submitted version.

## Conflict of Interest

The authors declare that the research was conducted in the absence of any commercial or financial relationships that could be construed as a potential conflict of interest.
